# Drug-resistant tuberculosis in Mumbai, India: An agenda for operations research

**DOI:** 10.1016/j.orhc.2012.06.001

**Published:** 2012-06

**Authors:** Nerges Mistry, Monica Tolani, David Osrin

**Affiliations:** a The Foundation for Medical Research, 84A RG Thadani Marg, Worli, Mumbai 400 018, Maharashtra, India; b Center for International Health and Development, UCL Institute of Child Health, 30 Guildford Street, London WC1N 1EH, UK

**Keywords:** Tuberculosis, Public health, Global health

## Abstract

Operations research (OR) is well established in India and is also a prominent feature of the global and local agendas for tuberculosis (TB) control. India accounts for a quarter of the global burden of TB and of new cases. Multidrug-resistant TB is a significant problem in Mumbai, India’s most populous city, and there have been recent reports of totally resistant TB. Much thought has been given to the role of OR in addressing programmatic challenges, by both international partnerships and India’s Revised National TB Control Programme. We attempt to summarize the major challenges to TB control in Mumbai, with an emphasis on drug resistance. Specific challenges include diagnosis of TB and defining cure, detecting drug resistant TB, multiple sources of health care in the private, public and informal sectors, co-infection with human immunodeficiency virus (HIV) and a concurrent epidemic of non-communicable diseases, suboptimal prescribing practices, and infection control. We propose a local agenda for OR: modeling the effects of newer technologies, active case detection, and changes in timing of activities, and mapping hotspots and contact networks; modeling the effects of drug control, changing the balance of ambulatory and inpatient care, and adverse drug reactions; modeling the effects of integration of TB and HIV diagnosis and management, and preventive drug therapy; and modeling the effects of initiatives to improve infection control.

## 1 Introduction

*Operations research, India, and tuberculosis*

India has operations research (OR) history. Established in 1957 and affiliated with the International Federation of Operational Research Societies (IFORS), the Operational Research Society of India was the first in the South, and hosted the first international conference on OR for Development, in Ahmedabad in 1992 (ORSI: www.orsi.in). Nevertheless, OR on public health has been limited, and in this India is no exception [1,2]. In a recent discussion, Royston suggests that OR has a rather broader interpretation in global health than in management science [3]. Health initiatives have tended to use group methods of qualitative character (brainstorming, behavioral simulation, scenario analysis, system mapping), rather than quantitative exercises such as mathematical modeling and system dynamics. We think this is true, but understandable. Proponents of OR for global health–and for TB control in particular–see it as research “intended to provide managers, administrators, and policy makers with the information that they need to improve service delivery activities and plan future ones” [4]; or, “research into strategies, interventions, tools or knowledge that can enhance the quality, coverage, effectiveness or performance of the health system or programmes in which the research is being conducted” [5,6]. This sort of language is less intimidating than “an interdisciplinary branch of applied mathematics or formal science that uses advanced analytic methods to make better decisions or research that provides optimal solutions to complex decision-making” (http://www.scienceofbetter.org/what/index.htm).

It turns out that OR for health in low-income countries has been championed by people involved in tuberculosis (TB) control, from making the case for OR [5,7–9] to more tightly specified agendas [10,11]. The sort of OR that might be described as implementation science is crucial to the success of TB programmes [12,13], and guided the development of current approaches based on Directly Observed Therapy Short-course (DOTS) [11]. Substantial efforts have been made to support OR for TB control through funding, training and guidelines, but it is not always clear that it has delivered [11]. Commentaries call for greater coordination — for activity to be more programme-driven than researcher-driven — [9] and for a clear sequence of steps from research to implementation [8].

The current WHO Stop TB Strategy has six concerns: (i) high-quality management of TB through DOTS, (ii) co-infection with TB and human immunodeficiency virus (TB-HIV), multidrug-resistant TB (MDR-TB) and the needs of poor and vulnerable populations, (iii) health systems, (iv) multiple providers of health care, and (v) partnerships with people with TB [14]. The sixth concern is the need for research, and the Global Plan to Stop TB 2011–2015 is unusual in emphasizing OR (http://www.stoptb.org) [15].

*Tuberculosis in India*

Nowhere is the OR agenda more pressing than in India, which accounts for 25% of the global burden of TB and 29% of global TB mortality [16]. TB causes an estimated 320 000 annual deaths in India: 17.6% of communicable disease deaths and 3.5% of all-cause mortality [17]. While the incidence has fallen from 216 to 185 per 100 000 over the last decade, notifications reached 1.5 million in 2010. 1.2 million of these were new cases. India contributed 23% of new diagnoses of TB globally, but 43% of individuals who required retreatment. The primary notional provider of care is the Revised National TB Control Programme (RNTCP): Fig. 1 is an overview of major activities.

*Mumbai*

If one wanted to design an environment conducive to the spread of TB, failure of treatment, and emergence of resistance, Mumbai would fit the remit. A land-starved megacity in which a resident population of 12.5 million are compressed into 437 km^2^[18], Mumbai combines extreme population density with inadequate water, sanitation, and solid waste management, indoor and outdoor air pollution, an unbridled multiplicity of healthcare providers, inequalities startling in their visibility and proximity, and an epidemic of predisposing malnutrition and illness. The city has a mean population density of 49 000 per km^2^[19], over half of whom live in the informal settlements, *zopadpattis, bastis* and *chawls* loosely described as slums [18]. TB is a disease of poverty and marginality [20], and the UN-HABITAT definition of a slum could double as a pro-tuberculotic checklist: overcrowding, inadequate safe water and sanitation, poor housing fabric, and insecurity of tenure [21]. Coupled with this are epidemics of non-communicable disease: 34% of women have low body mass index, and 44% are anaemic, and 46% of children in the poorest quartile are underweight [22]. Tobacco and alcohol use are common, and diabetes — which affects an estimated 40 million Indians [23] — has a prevalence of 1900 per 100 000 in slum-dwelling men and 1170 in women.

Mumbai houses 12% of the population of Maharashtra state, but accounts for 22% of notified cases of TB and — significantly in terms of potential drug resistance — 50% of people undergoing retreatment after relapse (SHRC, unpublished data). TB treatment is estimated at 600 per 100 000 in slum areas and 458 in non-slum areas [19,22]. The RNTCP registered ∼30 000 people for treatment in 2010, of whom ∼15 000 were smear-positive and ∼2000 (9%) were children. Given that 50%–70% of people seeking treatment do so in the private sector [24], the incidence of TB in Mumbai is likely to be at least 60 000 cases annually; half of them documented in terms of type, treatment, and outcome.

The emergence of drug-resistant TB in Mumbai is a prospect so alarming that the paucity of available evidence may be a case of ‘out of sight, out of mind’. What reports there are have consistently shown higher levels of MDR-TB (resistant to the 2 first-line drugs rifampicin and isoniazid) than in other parts of India, at 24%–30% of new cases [25,26] and 11%–67% of treated cases [25–27] (the corresponding figures from other parts of the country are 1%–13% and 12%–40%, respectively [28–35]). Extensively drug-resistant (XDR) TB (MDR-TB that is also resistant to both a fluoroquinolone and a second-line injectable agent) was detected in Mumbai in 2005 [36], but the most nightmarish of scenarios has been evoked by a recent report of totally drug resistant (TDR) TB [37].

In the fallout from the TDR-TB findings, which came from Hinduja Hospital and were widely reported [38], the local authorities have made a series of proposals. Key among them are the establishment of MDR-TB as a notifiable disease and the introduction of Xpert MTB/RIF^®^ (an automated nucleic acid amplification test) to facilitate rapid, quality drug susceptibility testing (DST). DOTS Plus regimens will be provided to clients failing first-line treatment by the public sector. More general proposals include increased human resources (the designation of each of the 24 municipal wards as a ‘TB district’, such that each would have its own TB control officer), training for healthcare cadres, improved infection control at TB hospitals (though not currently at peripheral facilities), and house-to-house visits and surveys in vulnerable areas. A combination of public–private mix models and efforts to control prescribing practices is proposed, with the idea that pharmacists’ associations might be involved in controlling over-the-counter sale of anti-TB medication.

## 2 Existing recommendations for operations research

Lienhardt and Cobelens categorize OR needs at three levels: improving programme performance and outcomes, assessing the feasibility, effectiveness or impact of new strategies or interventions on TB control, and collecting data to guide policy on specific interventions [11]. These map roughly onto local-or-national, national-or-regional, and regional-or-international tiers. There are serious concerns about TB control in Mumbai and we locate the OR needs on the local-national tier, clustered around programmatic issues: case-finding and diagnosis, ascription of drug sensitivity, delivery of appropriate treatment, follow-up, definition of cure or failure, and management of default (dropping out of the treatment programme), relapse, and drug resistance. These issues are by no means unique, but they are context-specific.

The Stop TB Partnership calls for OR under four general themes: (i) improving access, screening and diagnosis, (ii) sustainable collaboration with all care-providers, (iii) prevention of TB in people living with HIV, and joint treatment of HIV and TB, and (iv) access to and delivery of treatment. A fifth category applies specifically to OR [10]. India’s RNTCP has also developed detailed OR guidelines and an action plan, overseen by a central research cell, a national standing committee, and state-level OR committees. The agenda includes topics of primary and secondary importance, breaks them down in terms of whether they would best be addressed by research institutes, medical colleges, RNTCP units, or postgraduate students, and calls for proposals every six months [39]. Table 1 compares priority areas from both sets of guidelines.

## 3 Specific challenges for tuberculosis control in Mumbai

### 3.1 Improving access, screening and diagnosis of tuberculosis

*Diagnosing tuberculosis*

New cases entering the RNTCP are diagnosed by sputum microscopy, which is also used for follow-up. In principle, we do not know whether or not they represent MDR-TB (although, once diagnosed, treatment under the DOTS Plus regimen is monitored by sputum culture). It may also not be clear whether they are really ‘new’, or whether they have received treatment before. Misclassification has been reported in 13% of cases in Mumbai [40]. The reasons for this are both microbiological and systemic. Insufficient efforts to screen clients have been attributed to workload and negligence, even when clear guidelines were available. Clients may not have kept records of previous treatment, and may be reluctant to mention it for fear of either stigma or refusal to provide further treatment [40]. The target-oriented approach of the RNTCP may also predispose health workers to classify clients as new [41].

*Defining cure*

Microscopy performed on unconcentrated sputum samples has limited sensitivity, the concern being that it can lead to over-ascription of cure and a higher likelihood of relapse. Relapses constitute 38% of retreatment cases in India — six times as many as treatment failures. A study in Delhi found relapse in 9% of people who had been classified as cured [42], and over half of retreatment episodes occurred after RNTCP treatment [43]. The relatively good outcomes seen in three-quarters of these episodes suggest that the initial treatment might not have been optimal [42,44]. Levels of MDR-TB in treatment after relapse are 11%–27%, lower than after default, and lower in turn than after treatment failure [29,35,45].

*Detecting drug resistant tuberculosis*

India has 15 laboratories accredited for DST (RNTCP Performance Report, 2nd Quarter, 2011), much below the recommended population provision of one per 5 million. Most laboratories use solid culture, the turnaround time for which is 8–12 weeks. Three laboratories use line probe assays (LPA: nucleic acid amplification and tagging for resistance) that can provide results within a day. Of Mumbai’s 2 accredited laboratories, one uses liquid culture (turnaround 4 weeks) and the other — recently accredited — LPA. The process of coordination between these facilities in handling the city’s MDR-TB load is unclear.

### 3.2 Sustainable collaboration with all care-providers for tuberculosis control

*Choice of provider*

India’s private sector provides 70%–80% of outpatient consultations, almost 70% of hospitals and 40% of hospital beds [46–48]. With 50 large private hospitals, 2000 nursing homes, and 8000–10 000 individual private practitioners [49], Mumbai offers a wide range of medical services to its inhabitants [50,51], many of them an amalgam of allopathic and traditional medicine [52]. For TB control, this represents what Babu and Laxminarayan describe as a poverty of plenty [53].

Studies on care-seeking suggest that about half of people with chest symptoms make a first approach to a government facility, but the preference for private sector health care has been well described. The pull and push factors are familiar: proximity and a perception of better quality of care, underscored by dissatisfaction with public sector service timings, crowds and waiting times, staff availability, quality and attitude [54–56]. Women seem more likely than men to see private practitioners for an initial consultation for a cough [57], and the stigma of TB may make them more reluctant to undergo sputum examination [58]. Parents may conceal the fact that their daughter has TB because of concerns about her marriage prospects [59].

Although choice of health care may be influenced by awareness of available services [60], financial constraints are the major reason for opting for treatment from government facilities [54,55,61]. Cost may not be a threshold factor, but it is a limiting factor [22]. Awareness of the availability of free medicines at government facilities does not necessarily encourage uptake [62], and clients only move from private to public sector when the duration of their illness and the costs associated with long-term private care begin to bite [54]. ‘Treatment shopping’ has often been cited as a reason for delay in approaching the RNTCP [54]; understandable, perhaps, in a market in which people who stick with a single provider end up spending more [62]. One study estimated the average number of providers seen before approaching the RNTCP at 2, although this correlated more with the inability of providers to correctly diagnose TB than with client-driven shopping [54].

*Public–private mix*

The RNTCP has undertaken an extensive programme of collaboration with private and non-government practitioners. With the institution of referral processes, clarification of roles, and amendments to recording systems, the collaboration increased diagnoses of new smear positive TB by 40% [49]. Public–private mix projects have shown promising cure rates in other cities [63]. Clients in Delhi seemed to be satisfied with the arrangements, although the default rate was 14% [64]. There were also savings to the RNTCP because of the contribution of private sector facilities and human resources [65]. This contribution is, however, a potential limit to sustainability. Private providers, many of them single-handed and at the edge of profitability, often balk at the human resources and support systems necessary for monitoring, tracing defaulters, and record keeping [66].

### 3.3 Prevention of TB in people living with HIV, and joint treatment of HIV and TB

*Co-infection with HIV*

For Indians infected with Human Immunodeficiency Virus (HIV), the chance of developing active TB is 50%–60%, compared with 10% in the general population [67,68]. The prevalence of HIV is 0.34%, but the distribution is uneven [69], and this is reflected in HIV seroprevalence in people with TB. The national prevalence in notified TB infections is 5%–8% [70–72], with a variation of between 0.5% and 13.5% depending on location [73–77]. This poses operational challenges for the design and implementation of collaborative TB and HIV interventions [76].

Currently, 49% of people in India known to have HIV infection receive antiretroviral therapy (ART). The figure for Mumbai is 35% [72]. Death during TB treatment in HIV infected individuals is strongly associated with the absence of ART, independent of gender and previous TB treatment, which implies that programmes should promote high levels of ART uptake and monitor progress closely [77]. Treatment of TB in the presence of HIV is complex in terms of regimen duration, dosage and frequency of anti-TB drugs, optimal timing of initiation of ART, and optimal drug combination for people on second-line treatment [78]. In a study of clients on ambulatory treatment for MDR-TB-HIV in a slum setting in Mumbai, cure rates were 31% and treatment completion 17% at 2 years [79]. The relative success of community-based care was attributed to a one-stop service for TB and HIV, early management of adverse drug effects, psychosocial support, and administration of drug combinations based on DST results.

*Diabetes*

Among the epidemics that India finds itself facing is rampant diabetes mellitus. Case-control studies suggest odds ratios for developing TB of 2.4–8.3 in the presence of diabetes [80–84]. Smear positive TB was seen in 6% of diabetic clients in a study in eastern India [85], and diabetes was the most common risk factor for pulmonary TB in south Indian studies [81,86]. Mathematical models suggest that it accounts for 15% of pulmonary TB and 20% of smear-positive (infectious) TB in India [87]. Annual cases of TB in people with diabetes increased by 46% between 1998 and 2008 [88], and diabetes is associated with a 15% greater incidence of smear-positive TB in urban than in rural areas [87]. Modeling also suggests that the incidence of TB would have increased (by 28%) faster than population size (at 22%) because of the effects of aging, urbanization, changing body mass index and rising diabetes prevalence [87]. It is not certain whether people with diabetes are more likely to develop MDR-TB [89–93], although the levels are substantial in Indian clinics [94,95]. Diabetes may also exacerbate the hepatotoxicity of anti-tuberculous drugs [96].

### 3.4 Access to and delivery of treatment for drug-susceptible and M/XDR-TB

*Prescribing practices*

That treatment practices in the private sector are largely unregulated has been an issue for years [97]. Inappropriate TB regimens have been documented in the majority of indigenous medical practitioners [98], private practitioners in a slum area [24], and also in chest physicians [99]. A study in Delhi suggested that when health workers thought that patients were poor, marginalized, or itinerant, they might not begin DOTS because of the assumption that they would not adhere [100].

*Spurious drugs*

Use of substandard medications is analogous to inadequate therapy and could lead to drug resistance and treatment failure, despite 100% adherence [101]. An analysis of single and fixed-dose combinations of isoniazid and rifampicin available in India found 13% to be substandard, and the mean content of 100 samples of rifampicin purchased from pharmacies in Chennai was below pharmaceutical standards [102,103].

*The problem of default*

Dropping out of a treatment programme — and increasing the risk of development of MDR-TB — is the result of an interplay of client and provider factors. Key predispositions include a combination of relief from symptoms, the need to work, and stigma. Side-effects of treatment were cited as a reason for default by 10%–50% of defaulters [58,104,105], and default is commoner in men and alcohol users [104–106]. Migration during the extended period of treatment is clearly an issue, as are distance to services, DOTS service opening times, the need to travel while unwell, and costs of travel (three times a week in the case of DOTS) [104,106]. It is possible that DOTS provision by local generalists such as *anganwadi* workers is less liable to default than provision by specific DOTS centers, possibly because of proximity and ease of access, and possibly because of a less overt labeling of the problem as TB [58]. People who have defaulted previously, or who have been treated previously outside the RNTCP system, are more likely to default [104,105,107]. Provider-related issues include poor communication and lack of attention and support from health workers, especially when patients experience side-effects [104].

The peak time for default is at the end of the intensive phase and beginning of the continuation phase of treatment [105]. When clients default, healthcare workers are responsible for tracing them. These clients, however, may be labeled as ‘unlikely to comply’ and likely to ‘spoil the results of a TB center’. They may be treated insensitively when they return for care, or asked to produce a guarantor that they will complete the course [100,104].

*Infection control and transfer of resistance*

Infection control is a relatively neglected aspect of TB — particularly MDR-TB — prevention. With its high caseloads, Mumbai faces overwhelming numbers of clients in conditions in which repeated mutual exposure is common. Respiratory isolation wards, adequate ventilation and sunlight, and availability of N95 respirators are limited in clinical settings, and the provision of TB clinics within maternity care facilities, schools, and residences introduces a worrying level of unnecessary exposure to vulnerable groups. Nosocomial transmission affects both clients and health workers [108], and has been associated with outbreaks such as that seen in USA in the 1990s [109]. Working in health care is a risk factor for latent TB, amplified by delays in diagnosis and characterization [110–113]. In an endemic setting the tools to detect latent infection and differentiate it from exposure are, however, limited [113]. The annual risk of infection is 1.5% in the general population [114], 1.7% in young doctors in Delhi [115], and 4%–8% in nurses [110,112]. Recent media reports suggest substantial numbers of infections over the last 3 years in staff at Mumbai’s TB hospital, around half of infections leading to death. Unfortunately, the view of TB as an occupational hazard may limit efforts to prevent transmission.

For people visiting health posts for treatment, the risk of cross-infection appears to be high. In a Mumbai study of previously untreated clients with pulmonary TB, 32% of those who began with a sensitive or mono-resistant profile had developed MDR-TB by the fifth month. The fingerprint of 30% of paired isolates changed between the first and fifth months, suggesting new infection that might have been transmitted at health care facilities (unpublished data).

## 4 Four suggestions for operations research

A recent meeting convened by the Foundation for Medical Research brought together experts in an attempt to clarify the agenda for action and research (Focusing on solutions for TB control in Mumbai, February 2012). The broad recommendations of the meeting are summarized in Table 2. To be honest, we find the breadth of challenges and the existing prescriptions for OR intimidating. It is part of the function of the Stop TB Partnership and the RNTCP to take a broad view of TB control, and we add no local value to the process if we do not contribute fairly clear opinions. We therefore set ourselves the task of identifying four topics for OR for TB control in Mumbai. We tried to think outside the methodological comfort zone of knowledge, attitude and practice studies, and studies of client and provider perceptions, toward quantitative studies and more speculative modeling.

### 4.1 Better diagnosis, DST, classification and definition of cure

An example of the potential of existing records as modeling material comes from a study from Andhra Pradesh [116]. The RNTCP only conducts DST for clients judged to be at high risk of MDR-TB. The study looked at the proportion of clients who were judged to be ‘MDR suspects’, the proportion who ended up with culture and DST, the proportion diagnosed with MDR-TB, and the proportion who started appropriate treatment. Two percent of 24 000 clients were judged as being at risk of MDR-TB and 55% of them had culture. There were substantial losses between sputum collection, laboratory receipt, culture and DST results, and subsequent treatment.

*Modeling the effects of new technologies*

We think that fairly simple modeling would be useful in predicting the effects of new approaches on population incidence and prevalence. For example, there is justifiable current enthusiasm for introducing Xpert MTB/RIF^®^, which may be cost-effective for tuberculosis control in low-income countries [117,118]. Operations research models that factor in the likely losses on the pathway between symptom and cure would help us to identify pressure points in the systems that would need to surround the technology. It would also be helpful to model the effects on transmission rates of newer technologies for early diagnosis of TB in children.

*Mapping and network modeling*

Technologies such as geographic information systems (GIS), combined with infectious disease modeling, are potentially useful in identifying transmission hotspots. We would like to see analytics used in this way — along with DST — to answer questions about acquisition of MDR-TB at health facilities and in communities [13].

*Using active case detection to improve allocation of resources*

The identification of MDR-TB in Mumbai is a passive exercise. Countries have initiated active surveys for identifying TB in areas with a high burden of drug resistance. Two approaches to intensifying identification have been recently recommended to the RNTCP. The first is compulsory notification of MDR-TB (and, now, TB in general). The second is to carry out an active survey in vulnerable populations such as informal settlements and institutions. These approaches should link detected cases with treatment and follow-up. Modeling would be useful in estimating the projected reduction in transmission, and would allow realistic planning for drug requirements and a more reliable computation of costs.

*Modeling the effects of changes in timing of activities*

Current RNTCP guidelines recommend DST for patients who fail any treatment regimen. It would be useful to assess the appropriate time for DST. Would it be effective, for example, to act on early indicators of MDR-TB such as smear positivity in the second or third months of treatment, in the absence of other risk factors? How would providing DST results to clients on the same day affect incidence and prevalence, given the potential reduction in attrition? And what would be the population effects of introducing better tests at the end of treatment to confirm cure or predict potential relapse?

### 4.2 Better prescribing practices across care providers

*Modeling the effects of legislation and drug control*

Irregular prescription practices by a variety of health care providers are well described. We need to get the right drug combinations, in the right regimens, to more clients [119,120]. One option for OR is to examine the reasons for suboptimal management, particularly by private and informal sector providers, and particularly the use of fixed-dose combinations whose curative efficacy is uncertain. India is currently developing policies to regulate access to anti-TB drugs, and it would be interesting to model the potential effects of changes in availability across a range of scenarios. Sensitivity analyses might include increased use of the RNTCP, reduction in treatment per se, and unauthorized prescription.

*Modeling the effects of changing the balance of ambulatory and inpatient management*

The WHO guidelines for treatment of MDR-TB recommend predominantly ambulatory care over hospitalization [120]. Benefits have been demonstrated in one Mumbai study [78], but need to be assessed in settings with different constellations of risk factors. Models could include risks of non-adherence, relapse and default, as well as risks of acquisition at facilities and in the community.

*Modeling the effects of adverse drug reactions*

Pharmacovigilance — including systematic surveillance of drug-related problems — is needed because relatively little attention is paid to the adverse reactions that can precipitate default [119]. Models describing the potential effects of this default on TB control would help the RNTCP to counsel clients, improve adherence, and retain public confidence.

### 4.3 Better integration of TB diagnosis and treatment with ART for HIV

*Modeling diagnosis and transmissibility*

The National Framework for Joint HIV/TB Collaborative Activities identifies priority areas for OR [121]. In addition to the RNTCP and crossover recommendations on programmatic OR (Table 1), it recommends the development of an algorithm to exclude active TB in ART clients. Models that include a range of factors are intrinsic to the development of potential algorithms and to fine-tuning their utility in terms of positive and negative predictive value. We also need contact data in order to build epidemiologic models that address the transmission of TB to people living with HIV, and from them to uninfected individuals.

*Modeling the effects of isoniazid preventive therapy*

The National AIDS Control Organisation (NACO) acknowledges the international evidence for a role for isoniazid preventive therapy (IPT: giving isoniazid to ART clients to prevent the development of TB). Although the national guidelines recommend tests of the feasibility and cost-effectiveness of IPT, it has not yet been adopted because of concerns about feasibility of implementation and efficacy within India’s ART programme. A rapid feasibility and efficacy study has been proposed by NACO [122]. High levels of isoniazid resistance have been recorded [123], and scenario modeling would help to clarify the population effects of a potential programme at scale. IPT has also been suggested for child contacts of TB patients [124], and with a current focus on the diagnosis and prevention of childhood TB, evaluating the efficacy of IPT in children would be useful.

### 4.4 Better infection control

*Modeling the effects of changes to infrastructure and routines*

DOTS Plus regimens for MDR-TB require both outpatient and inpatient care and the resulting risk of transmission must be offset by appropriate infection control measures. Particular issues are the design of health posts and wards and the need for distinct time-slots for different categories of clients given the integration of TB care with activities such as maternity care and immunization. Systems to fast-track smear positive individuals need to reflect the caseloads at different facilities. Models could help us to understand the transmission dynamics (especially of M/XDR-TB) within facilities, and the likelihood of subsequent transmission in the community [125].

## 5 Summary

The emergence of multi- and totally drug resistant TB in Mumbai presents health workers and policy makers with an urgent, multifaceted problem. In identifying an agenda for operations research to assist in tackling the challenges, we have highlighted areas in which improved analysis of data and the development of models focused on specific aspects of policy and health service delivery could beneficially inform decisions on the ground. We encourage operations researchers to engage with academics, practitioners and policy makers working in global health to respond to this agenda.

## Figures and Tables

**Fig. 1:**
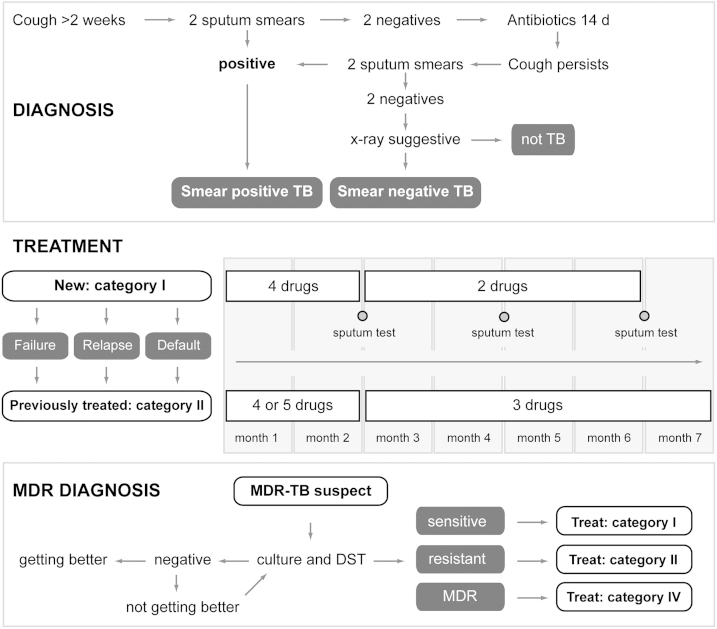
Schematic overview of key tuberculosis control activities in India’s Revised National Tuberculosis Control Programme.

**Table 1:** Recommendations for operations research, adapted from Stop TB Alliance [10] and RNTCP guidelines [39].

Stop TB OR recommendations	RNTCP immediate priority areas for OR
**Improving access, screening and diagnosis of TB**	**Interventions to improve case detection and diagnosis**
How to improve access to TB diagnosis?	Care seeking behavior and reasons for diagnostic delay in vulnerable populations, including urban slum dwellers.
How to improve screening of TB suspects and high-risk groups?	Pilot test of “ 2+2” (2 weeks cough and 2 sputum specimens) for TB suspect identification and diagnosis in high and low workload settings.
How to use the introduction of new tools to improve service delivery practices?	Yield of sputum-smear examination for extrapulmonary TB at diagnosis, and predictive value of follow-up sputum-smear examination in extrapulmonary and smear negative TB.
How to improve active TB case-finding?	Prevalence of cough >2 weeks among outpatients, and smear microscopy outcomes among them.
How to build accessible, effective and efficient diagnostic services with new diagnostic tools?	
How to evaluate the impact of new tests or new approaches?	
**Prevention of TB in people living with HIV, and joint treatment of HIV and TB**	**Interventions to address TB/HIV**
What are the barriers to TB diagnosis, and how to overcome them?	Evaluation of screening methods for TB case-finding in antiretroviral treatment, HIV care and support centers.
What are the barriers to initiation of isoniazid preventive therapy?	Reasons for loss of TB suspects referred from integrated counseling and testing centers to microscopy centers.
What are the barriers to optimal combined TB/HIV diagnosis and treatment, and what are the optimal models for joint TB and HIV care activities?	Reasons for non-initiation of ART for clients with TB and HIV.
	Incidence and mortality associated with TB among clients awaiting ART and on ART.
**Access to and delivery of treatment for drug-susceptible and M/XDR-TB**	**Interventions to address drug-resistant TB**
What are the drivers of drug-resistant TB at individual and programmatic levels?	Prevalence of MDR-TB in Category I failures, Category II entry, and Category II clients smear positive at 3 months, and association of MDR-TB with source of and past history of anti-TB treatment.
What are the potential strategies for integration/scale up of drug-resistant TB management within TB control programmes?	Evaluation of innovative methods of community-based DOT provision for the delivery of RNTCP Category IV treatment.
	Rapid case-control study for risk factors for fluoroquinolone resistance and XDR-TB among clients with MDR-TB.
	Use of second-line anti-TB drugs and MDR-TB diagnostic and treatment practices among providers in urban areas (surveys).
**Sustainable collaboration with all care-providers for TB control**	**Interventions to engage all health care providers**
How to improve and scale up existing approaches to engaging all care-providers?	Evaluation of the quality of TB diagnosis and care among private sector physicians.
How to measure the contribution of different provider groups to TB care and control?	Marketing to private health providers: what messages change referral, diagnostic, and treatment behavior for TB?
How to encourage involvement of as yet unengaged providers?	Evaluation of comparative results and effort required by the different RNTCP schemes to involve private practitioners.
How to encourage involvement of the non- public sector in MDR-TB management and TB/HIV collaborative activities?	Knowledge, attitudes and practices of providers of alternative systems of medicine.
How to develop and assess responses to changing involvement of diverse providers in TB care and control?	Testing methods to involve providers of alternative systems of medicine in the referral of TB suspects.
How to encourage introduction of regulatory approaches to collaborating care-providers?	
**Access to and delivery of treatment for drug-susceptible and M/XDR-TB**	**Improving community access to TB services**
What are the reporting gaps and deficiencies in first-line management of TB cases?	Qualitative (focus groups) and quantitative (pre-and post intervention) evaluation of the effectiveness of communication methods and messages to promote client demand.
How to address these deficiencies and improve management of drug-sensitive TB?	Testing innovative interventions to increase public visibility of TB diagnosis and treatment facilities.
How to reinforce PPM collaboration for treatment of sensitive- and resistant TB?	Efficacy and cost of innovations to increase demand by people with respiratory symptoms in primary care.
How to improve decentralized and fully integrated access to TB and antiretroviral treatment?	
	**Interventions to improve treatment outcomes**
	Prospective, community-based, long-term cohort study of RNTCP clients:
	Risk factors for death, default, and treatment failure. Impact of migration on treatment outcomes. Impact of diabetes and HIV infection on treatment outcomes. Impact of non-MDR drug resistance on treatment outcomes. Incidence and risk factors for relapse and reinfection. Risk factors for death after treatment.
	Evaluation of reasons for initial default and effectiveness of interventions to prevent it.
	Cluster randomized controlled trial of innovative and cost-effective programme interventions to reduce default.
	Impact on outcome and relapse of daily or partially intermittent treatment (2 weeks daily) during the intensive phase of TB treatment, compared with fully-intermittent regimen, in clients with and without HIV infection.

**Table 2:** Recommendations of the expert group, *Focusing on Solutions for TB Control in Mumbai*, 2012.

Active survey, notification and surveillance of MDR-TB cases.
Recognition of stigma and dealing with its elimination; guaranteeing client confidentiality.
Strengthening client counseling services.
Social support for clients.
Prevention of drop-out; increasing the role of civil society in preventing drop-out.
Increase in drug sensitivity testing and diagnostic laboratories.
Consistent and continuous supply of drugs.
Integration of the health system for TB and co-morbidities such as diabetes.
Implementation of existing RNTCP protocols related to client handling and infection control.
Preventing misuse of drugs by implementation of existing rules and regulations pertaining to over-the-counter sale.
Increased OR within the system. Facilitation of collaborative research projects to increase analytical capacity and introspection by the RNTCP.
Formation of a Technical Advisory Group, taking into consideration the available expertise in the city for surveillance and control measures.

## Appendix Supplementary data

MMC S1 DOTS services poster at Urban Health Center, Dharavi, Mumbai, 2012.
